# Assessing the Impact of COVID-19 Vaccination on Preterm Birth: A Systematic Review with Meta-Analysis

**DOI:** 10.3390/vaccines12010102

**Published:** 2024-01-19

**Authors:** Mihaela Uta, Marius Craina, Felicia Marc, Ileana Enatescu

**Affiliations:** 1 Discipline of Obstetrics and Gynecology, “Victor Babes” University of Medicine and Pharmacy, 300041 Timisoara, Romania; uta.mihaela@umft.ro (M.U.); craina.marius@umft.ro (M.C.); 2 Doctoral School, “Victor Babes” University of Medicine and Pharmacy, 300041 Timisoara, Romania; 3 Department of Medical Sciences, Faculty of Medicine and Pharmacy, University of Oradea, 410073 Oradea, Romania; 4 Discipline of Neonatology, “Victor Babes” University of Medicine and Pharmacy, 300041 Timisoara, Romania; enatescu.ileana@umft.ro

**Keywords:** COVID-19, vaccination, SARS-CoV-2, pregnancy

## Abstract

During the coronavirus diseases 2019 (COVID-19) pandemic, the safety and efficacy of vaccination during pregnancy, particularly regarding the risk of preterm birth, have been a subject of concern. This systematic review aims to evaluate the impact of COVID-19 vaccination on preterm birth risk and to inform clinical practice and public health policies. Following PRISMA (Preferred Reporting Items for Systematic Reviews and Meta-Analyses) guidelines, a database search included PubMed, Embase, and Scopus, conducted up until October 2023. Inclusion criteria focused on studies that examined COVID-19 vaccination during pregnancy and its correlation with preterm birth, defined as a birth before 37 weeks of gestation. Six studies met these criteria, encompassing 35,612 patients. A quality assessment was performed using the Newcastle–Ottawa Scale and the Cochrane Collaboration’s tool, with the risk of bias evaluated via a funnel plot analysis and an Egger’s regression test. The studies demonstrated geographical diversity, mainly from Israel, Romania, and the United States, with a blend of prospective and retrospective designs. The patient cohort’s mean age was 31.2 years, with common comorbidities such as gestational diabetes and obesity affecting 9.85% of the total population. The vaccination types varied across the studies, with BNT162b2 being the most used. The results indicated a low heterogeneity among the included studies, evidenced by a Cochran’s Q statistic of 2.10 and an I^2^ statistic of 13%. The meta-analysis yielded a pooled odds ratio (OR) for a preterm birth risk post-vaccination of approximately 1.03 (95% CI: 0.82–1.30), suggesting no significant increase in preterm birth risk was associated with COVID-19 vaccination. Notable findings included a low preterm birth rate (as low as 0.6% and up to 6.1%) with minimal differences in neonatal outcomes, such as birth weight and APGAR (appearance, pulse, grimace, activity, and respiration) scores between vaccinated and unvaccinated groups. This study concludes that a COVID-19 vaccination during pregnancy does not significantly increase the risk of preterm birth. These findings are crucial for reassuring healthcare providers and pregnant women about the safety of COVID-19 vaccines and supporting their use in public health strategies during the pandemic.

## 1. Introduction

The (Severe acute respiratory syndrome coronavirus 2) SARS-CoV-2 virus, responsible for the coronavirus disease 2019 (COVID-19) pandemic, has significantly impacted public health worldwide [1,2], which extends to special population groups, such as pregnant women, where the interplay of viral infection and vaccination raises critical clinical questions [3,4]. The physiological changes during pregnancy alter immune responses and may affect susceptibility to infections and their outcomes [5], making the study of COVID-19’s impact on this demographic particularly vital [6].

Pregnant women with COVID-19 have been found to have an increased risk of severe illness and adverse pregnancy outcomes compared to their non-pregnant counterparts [7,8]. These outcomes prominently include preterm birth, a significant concern given its implications for neonatal health [9,10]. Studies have shown that a COVID-19 infection during pregnancy increases the risk of preterm birth by up to 50% higher than the non-infected counterparts [11], necessitating a deeper understanding of its pathophysiology, implications, and risk factors [12].

Conversely, the role of COVID-19 vaccination during pregnancy has been the subject of extensive research [13,14,15]. Studies have critically assessed the safety and efficacy of these vaccines in pregnant women. A significant finding from these studies is that COVID-19 vaccination during pregnancy does not increase the risk of adverse perinatal outcomes, rather, it appears to provide protective effects against complications such as intensive care unit admissions and maternal SARS-CoV-2 infection [16,17]. Thus, the potential of COVID-19 vaccination to reduce the risk of preterm birth is a critical area of focus. The interplay between maternal immunity, vaccine response, and neonatal outcomes underlines the need for targeted research in this domain.

The primary hypothesis of this study is that COVID-19 vaccination during pregnancy significantly reduces the risk of preterm birth. This review aims to analyze the efficacy of various vaccines in different populations, providing a comprehensive overview of their impact on maternal and neonatal health. The objective is to inform clinical practice and public health policies, enhancing the care and safety of pregnant women during the ongoing pandemic.

## 2. Materials and Methods

### 2.1. Protocol and Registration

This systematic review, conducted in October 2023, was focused on assessing the impact of COVID-19 vaccination on preterm birth. A systematic search was performed across three electronic databases including PubMed, Embase, and Scopus, with the literature scope extending up to October 2023. Our search strategy incorporated a diverse array of keywords and phrases specifically chosen to comprise the broad aspects of the study’s objective. These keywords included “COVID-19 Vaccine”, “SARS-CoV-2”, “Pregnancy”, “Preterm Birth”, “Vaccination During Pregnancy”, “Maternal Health”, “Neonatal Outcomes”, “Vaccine Safety in Pregnancy”, “Vaccine Efficacy in Pregnant Populations”, “Gestational Vaccination”, “Perinatal Health”, “Maternal-Fetal Immunity”, “Vaccination Timing During Pregnancy”, “Adverse Pregnancy Outcomes”, and “Maternal Immunization.”

The developed search strategy methodically combined these terms in various configurations to ensure a comprehensive retrieval of the relevant literature. The search strings used were: (“COVID-19 Vaccines” OR “SARS-CoV-2 Immunization”) AND (“Pregnancy” OR “Gestational Period”) AND (“Preterm Birth” OR “Premature Delivery”) AND (“Vaccine Safety in Pregnancy” OR “Vaccine Efficacy in Pregnant Populations”) AND (“Perinatal Health” OR “Maternal-Fetal Immunity”). The review adhered to the Preferred Reporting Items for Systematic Reviews and Meta-Analyses (PRISMA) guidelines [18] and the International Prospective Register of Systematic Reviews (PROSPERO) [19], ensuring a structured and transparent methodological approach. Additionally, this review was registered on the Open Science Framework, with the registration code osf.io/za4rs.

### 2.2. Eligibility Criteria and Definitions

In this systematic review, our focus was specifically on English-language journal articles that examined the relationship between COVID-19 vaccination during pregnancy and the incidence of preterm birth. Initially, we removed duplicate entries, followed by a detailed screening of abstracts by two independent researchers to ensure that each study was relevant to our research questions. Discrepancies in selection were resolved by consulting a third researcher.

The inclusion criteria for this review were defined as follows: (1) Studies that specifically investigated the impact of COVID-19 vaccination during pregnancy on the incidence of preterm birth; (2) Research that included pregnant populations vaccinated against COVID-19 and measured outcomes related to preterm birth; (3) Studies that provided a clear and detailed methodology regarding the assessment of vaccination status and timing during pregnancy; (4) Research that offered explicit details on how preterm birth was defined and assessed in the context of COVID-19 vaccination; (5) Research that included clinical trials, cohort studies, case-control studies, and case series.

Conversely, the exclusion criteria encompassed: (1) Studies not focused on the impact of COVID-19 vaccination during pregnancy on preterm birth outcomes; (2) Research that included non-pregnant populations or did not specifically analyze the link between COVID-19 vaccination and preterm birth; (3) Studies that did not provide clear outcome measures related to preterm birth; (4) Publications that were non-peer-reviewed articles, preprints, in-vitro studies, conference proceedings, general reviews, systematic reviews, meta-analyses, case reports, commentaries, and editorial letters.

Preterm birth, for the purpose of this review, was defined as any birth occurring before 37 completed weeks of gestation. This definition aligns with standard obstetric guidelines and allows for a consistent and clear understanding of the primary outcome measure across the included studies. COVID-19 vaccination was considered as any vaccination scheme from 1 to 3 doses of Pfizer (BNT162b2), AstraZeneca (ChAdOx1-S/nCoV-19), Moderna (mRNA-1273), or Janssen (Ad26.COV2.S) vaccines.

### 2.3. Data Collection Process

In this systematic review, the initial database search identified a total of 1233 articles. Of the total number of screened articles (281), 152 were identified as duplicates and removed. The remaining 156 articles underwent a preliminary screening based on abstracts, leading to the exclusion of non-relevant studies. This was followed by a thorough full-text review of the shortlisted articles by two authors, with disagreements resolved by a third author to ensure accuracy and objectivity.

**Figure 1:**
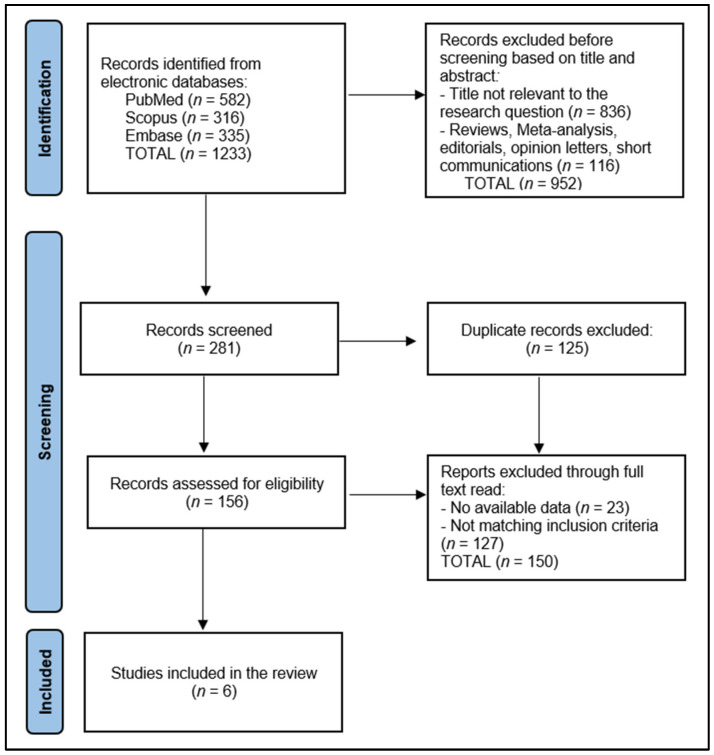
PRISMA Flow Diagram.

A total of 6 articles met the study protocol and were included in the review. The data extraction process, conducted by two researchers (M.U. and I.E.), involved gathering information on the study design, the participant demographics, the vaccine type and dosing, the timing of vaccination relative to COVID-19 infection, and the outcomes related to the post-COVID syndrome, as presented in Figure 1.

### 2.4. Risk of Bias and Quality Assessment

For quality assessment, we employed the Newcastle–Ottawa Scale for cohort studies and the Cochrane Collaboration’s tool for randomized trials [20]. Each study was independently evaluated by two researchers, with scores indicating the quality of the studies: low, medium, or high. This approach ensured an unbiased evaluation of the selected literature, forming the basis for our systematic analysis.

**Figure 2:**
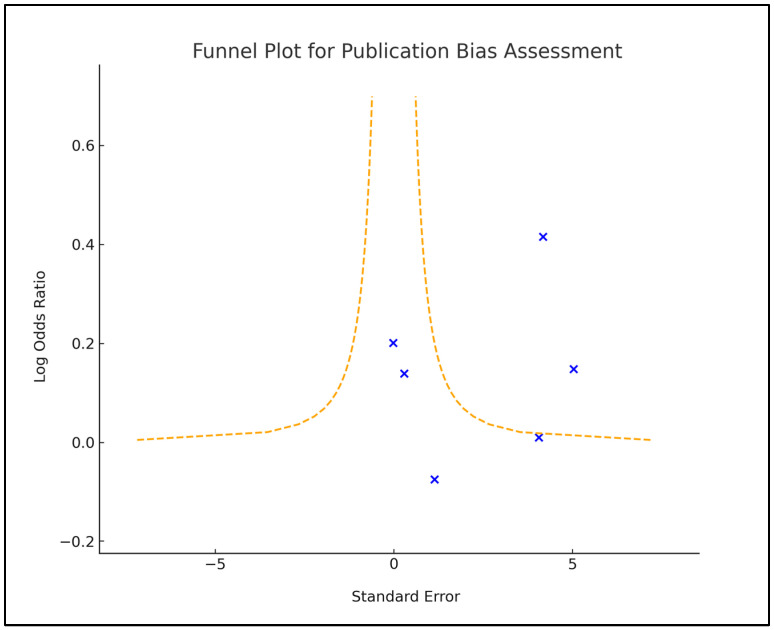
Funnel plot for publication bias.

A funnel plot was created to evaluate publication bias by plotting the effect sizes against their standard errors. The symmetry of the funnel plot was visually inspected and further analyzed using an Egger’s regression test, with a *p*-value < 0.05 indicating a significant publication bias. Additionally, we performed a sensitivity analysis to assess the robustness of the findings. This involved the sequential exclusion of individual studies from the analysis and recalculating the effect sizes, thereby determining the influence of single studies on the overall results, as seen in Figure 2.

### 2.5. Statistical Analysis Methods

In our systematic review, we conducted a meta-analysis using a random-effects model to evaluate the impact of COVID-19 vaccination on preterm birth risk. This model was chosen to account for potential differences among studies due to varying populations, methodologies, or vaccine types. The primary measure of the effect size was the odds ratio (OR), with risk ratios (RR) and hazard ratios (HR) also considered when available. We utilized Python software v.3.12.0 for data synthesis and the creation of forest plots, to display individual study findings and the overall pooled estimate of preterm birth risk post-vaccination. To assess the heterogeneity between studies, we calculated Cochran’s Q statistic and the I^2^ statistic.

## 3. Results

### 3.1. Study Characteristics

The systematic review analyzed a total of six studies [21,22,23,24,25,26], as shown in Table 1. These studies were geographically diverse, originating from Israel, Romania, and the United States. Israel contributed three studies to the review, with publications in 2021 and 2022 [21,24,25], reflecting a research interest in this area within the country. The United States also showed substantial interest, contributing two studies [23,26] to the review, both published in 2021 and 2022. Romania added one study [22], published in 2022.

The majority of the studies utilized a prospective cohort design [21,22,24], prevalent in half of the studies. The remaining studies adopted a retrospective cohort design [23,25,26]. In terms of study quality, one study was rated as ‘High’ [24], indicating a robust methodological approach and potentially more reliable findings. The remaining studies were rated as ‘Medium’ [21,23,25,26] or ‘Low’ [22].

### 3.2. Characteristics of Patients

The total number of patients across all studies was 35,612, with the smallest cohort in the study by Citu et al. [22], comprising 227 vaccinated and 608 unvaccinated patients, and the largest by Goldshtein et al. [24], involving a total of 24,288 patients (pre-post-inverse probability of treatment weighting (IPTW) and post-IPTW combined). The age range of the patients varied across studies, with the mean or median age generally hovering around the early thirties. For instance, Beharier et al. [21] reported a mean age of 28.8 years in the COVID-19 group and 31.7 years in the no-COVID-19 group, while Theiler et al. [26] noted a median age of 31.8 years in vaccinated patients and 30.1 years in unvaccinated patients.

A range of comorbidities was reported across the studies. Dick et al. [23] reported that 1.1% of patients had hypertensive disorders of pregnancy, and 9.6% had gestational diabetes. In contrast, Citu et al. [22] noted obesity rates of 19.6% in vaccinated patients and 22.4% in unvaccinated patients. The comparison groups typically included unvaccinated individuals, with some studies also categorizing patients based on their COVID-19 infection status or other characteristics. For instance, Beharier et al. [21] used a comparison group of 213 mother-child pairs, which included polymerase chain reaction (PCR)-positive, vaccinated, and unvaccinated noninfected patients.

Other characteristics noted in the studies included factors such as ethnicity, socioeconomic status, parity, and a history of previous medical treatments or conditions. For example, Rottenstreich et al. [25] reported that vaccinated women had higher rates of previous miscarriages and cesarean deliveries compared to unvaccinated women. In contrast, Goldshtein et al. [24] highlighted differences in socioeconomic status and population subgroups between vaccinated and unvaccinated groups, as presented in Table 2.

### 3.3. COVID-19 Vaccination Characteristics

The review highlighted a range of COVID-19 vaccines used across different studies. Specifically, Beharier et al. [21] and several others primarily used the BNT162b2 vaccine. In contrast, Citu et al. [22] and Theiler et al. [26] included a mix of the BNT162b2 and Ad26.COV2.S, and mRNA-1273 vaccines. Regarding dosage, most studies reported either one or two doses of the vaccine. For instance, Beharier et al. [21] administered two doses of BNT162b2, while Citu et al. [22] varied between one and two doses of BNT162b2 and Ad26.COV2.S. The timing of vaccination was also varied, with Beharier et al. [21] vaccinating at a median of 34.5 weeks GA and Theiler et al. [26] at a median of 32 weeks GA.

In terms of immune response, Citu et al. [22] reported a significant increase in spike antibodies post-vaccination, with levels rising from 0.41 U/mL to 1083 U/mL in seronegative individuals and from 145 U/mL to 10,759 U/mL in seropositive individuals within four months. The impact of COVID-19 vaccination on newborn features was a key focus. Citu et al. [22] reported average birth weights of 3149 g in vaccinated mothers versus 3207 g in unvaccinated ones. Additionally, the study noted APGAR scores of <7 at 5 min in 6.3% of newborns from vaccinated mothers compared to 6.6% from unvaccinated mothers. In contrast, Rottenstreich et al. [25] observed similar birth weights between vaccinated (3317.8 g) and unvaccinated (3339.5 g) groups, with comparable gender distribution and APGAR scores at 1 and 5 min. Goldshtein et al. [24] reported that 96% of newborns from vaccinated mothers were born at ≥37 weeks of gestation. Similarly, Theiler et al. [26] noted that 90.7% of deliveries were at ≥37 and 0/7 weeks, with a low birth weight occurrence of 7.9% and very low birth weight of 2.1%, as presented in Table 3.

### 3.4. Analysis of Outcomes

Across the studies, the pregnancy complications varied, with preterm birth rates ranging from as low as 0.6% in Beharier et al. [21] to 6.1% in Goldshtein et al. [24] (pre-IPTW unvaccinated group). Notably, Rottenstreich et al. [25] observed a 4.4% preterm birth rate, with additional complications such as elective cesarean delivery (11.5% in vaccinated vs. 7.6% in unvaccinated) and postpartum hemorrhage (7.3% in vaccinated vs. 10% in unvaccinated). Neonatal outcomes, such as Small for Gestational Age (SGA) and NICU admissions, were reported with slight differences between vaccinated and unvaccinated groups. For instance, Citu et al. [22] reported a 0.9% incidence of SGA in newborns, whereas Dick et al. [23] observed a slightly higher rate of SGA (6.2%).

The risk of preterm birth, assessed through the odds ratios (OR), risk ratios (RR), and hazard ratios (HR), showed a range of values across studies. Beharier et al. [21] reported an OR of 0.57 (0.16–2.06), suggesting no significant increase in preterm birth risk following vaccination. Similarly, Rottenstreich et al. [25] found an OR of 1.01 (0.62–1.63), indicating a neutral risk associated with vaccination. On the other hand, Dick et al. [23] found varying risks depending on the trimester of vaccination, with an OR of 1.49 (1.11, 2.01) for preterm birth in the second-trimester vaccinated group.

**Figure 3:**
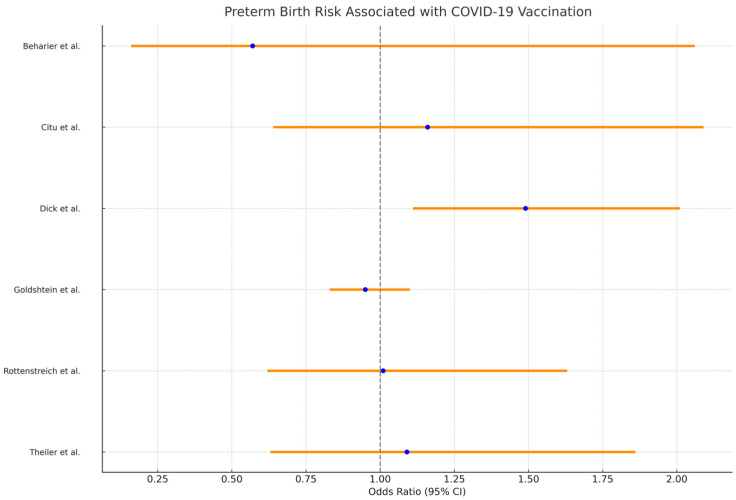
Forest plot analysis for the risk of preterm birth after COVID-19 vaccination during pregnancy [21,22,23,24,25,26].

Additional risks assessed included adverse pregnancy outcomes, SGA, and specific neonatal complications. For instance, Beharier et al. [21] reported an OR of 2.87 (0.33–25.09) for adverse pregnancy outcomes. Goldshtein et al. [24] noted a reduced risk of major heart malformations (OR 0.46 [0.24–0.82]) and Rottenstreich et al. [25] observed a significantly reduced risk of composite adverse neonatal outcomes with vaccination (OR 0.5, 0.36–0.74), as presented in Table 4 and Figure 3.

The meta-analysis of studies assessing the impact of COVID-19 vaccination on preterm birth risk demonstrated a pooled odds ratio of approximately 1.03. This value, however, suggests a marginal increase in the risk of preterm birth associated with COVID-19 vaccination. This inference is supported by the 95% confidence interval, which ranges from about 0.82 to 1.30, encompassing the null value and indicating a lack of strong evidence for a significant effect. Furthermore, the analysis revealed a notable lack of heterogeneity among the included studies, evidenced by a Cochran’s Q statistic of 2.10 and an I^2^ statistic of 13%, suggesting that there was little to no observed variation among the study outcomes that could be attributed to differences in the study design, populations, or other factors.

## 4. Discussion

This systematic review critically evaluated the implications of COVID-19 vaccination on preterm birth risk, encompassing a diverse array of studies from Israel, Romania, and the United States. The studies collectively involved a cohort size of 35,612 patients, and reported diverse comorbidities, reflecting the complexity and heterogeneity inherent in the studied populations, being different not only in terms of demographics but also in comorbid conditions, ranging from hypertensive disorders and gestational diabetes to obesity. The comparability groups, primarily consisting of unvaccinated individuals, provided a crucial context for evaluating the vaccination impact. However, variations in factors such as ethnicity, socioeconomic status, and medical history (e.g., previous miscarriages and cesarean deliveries) present in the studies underscored the multifaceted nature of the patient cohorts.

From a clinical perspective, the nuanced findings of this review have profound implications. While the pooled odds ratio pointed towards a slight increase in preterm birth risk associated with vaccination, the data did not robustly support a definitive causal relationship. This suggests that the decision to vaccinate during pregnancy, particularly considering the diverse vaccine types and dosages, should be tailored, taking into account individual patient profiles and risk factors. Moreover, the findings highlight the need for ongoing surveillance and research in this area, especially given the evolving nature of the pandemic and the introduction of new vaccines and booster doses. Ultimately, this review provides critical insights for informing clinical practice and public health policies, aiming to enhance the care and safety of pregnant women during the COVID-19 pandemic.

Evidence indicates that a SARS-CoV-2 infection during pregnancy heightens the risk of severe COVID-19 complications, including increased chances of hospitalization, ICU admission, mechanical ventilation needs, and mortality, compared to non-pregnant individuals. Pregnant patients also experienced higher rates of adverse outcomes such as preterm birth and stillbirth [12,27]. Given these risks, vaccination becomes vital for pregnant women to protect both themselves and their fetuses. Initially, vaccine trials did not include pregnant women, raising concerns about vaccine safety during pregnancy. Pregnant women, who are often more cautious about new vaccines, require comprehensive information to make informed vaccination decisions [28]. Additionally, maternal vaccination has been suggested to facilitate the transfer of neutralizing antibodies to the fetus, potentially granting neonatal immunity. This is crucial as neonates and infants are at a higher risk of severe COVID-19 illness, and currently, there are no approved vaccines for children under two years of age [29,30].

Pregnant women and their newborns are particularly susceptible to COVID-19, facing higher risks of illness and death compared to similar non-pregnant groups [31]. It has been reported that anti-SARS-CoV-2 antibody transfer to fetuses is notably reduced in cases of infection during the third trimester [32]. However, this study found that for infections occurring in the second trimester (weeks 15–30), there was a significant presence of maternal and cord-blood anti-COVID-19 antibodies at delivery, indicating efficient antibody transfer. This suggests that the diminished antibody transfer observed in late pregnancy might be due to delayed placental transfer.

Recent guidance recommends vaccination after a natural infection to boost immunity, yet its importance during pregnancy remains debated and lacks substantial evidence [33]. The study showed that women infected in the second trimester maintained strong immunity, indicating that titer testing before additional vaccination might be beneficial. Despite initial exclusion from vaccine trials, the urgent need for protection led to the inclusion of pregnant women in Israel’s vaccination campaign. This study reported a strong immune response in pregnant women vaccinated with the Pfizer-BioNTech mRNA vaccine, with effective antibody transfer to the fetus, surpassing that of third-trimester infections [34]. Moreover, the study found vaccine-induced antibodies in breast milk, suggesting another layer of infant protection. However, safety concerns still necessitate further research. Additionally, the study distinguished between immunity from infection and vaccination, finding asymptomatic, undiagnosed cases among vaccinated individuals and no evidence of a fetal IgM response to vaccine-induced antigens. This is contrary to some PCR-positive deliveries which indicated possible fetal exposure or vertical transmission, as indicated by other studies [35,36].

Nevertheless, the reviewed studies generally found no significant link between COVID-19 vaccination during pregnancy and adverse pregnancy outcomes. For instance, Rottenstreich et al. reported a lower rate of composite adverse neonatal outcomes in vaccinated mothers, although no differences were observed in individual neonatal outcomes between vaccinated and unvaccinated groups [25]. Magnus et al. noted a modest reduction in neonatal care admissions and low APGAR scores following third-trimester vaccination [37]. Moreover, the study reported an adjusted hazard ratio of 0.98 (95% CI 0.91–1.05). Conversely, Dick et al. observed an increased rate of preterm birth in women vaccinated during their second trimester compared to their unvaccinated counterparts [23]. Goldshtein et al. found that congenital malformation rates in vaccinated women were not higher than in unvaccinated women and aligned with pre-pandemic levels [24].

Our systematic review revealed significant limitations in the availability of such detailed data. Specifically, Goldshtein et al.’s study [24] reported on preterm births, distinguishing between early preterm births (less than 32 weeks), observed in 0.6% of unvaccinated and 0.4% of vaccinated individuals, and late preterm births (32–36 weeks), occurring in 3.5% of unvaccinated and 4.0% of vaccinated groups. Similarly, Rottenstreich’s study [25] indicated a 2.8% incidence of a gestational age at delivery of less than 34 weeks in both unvaccinated and vaccinated groups. Theiler’s study [26] further contributed to this categorization by reporting gestational ages at delivery, with 1.1% to 1.4% in the 24 0/7–31 6/7 weeks category, and a small percentage (0.2% to 0.7%) in the less than 24 weeks category. Future studies should report on the effect of COVID-19 vaccination on the severity of preterm birth. Another stratification worth reporting in future studies is the effect of vaccination on spontaneous and medically indicated preterm births.

The meta-analysis indicated a pooled odds ratio of approximately 1.03 for preterm birth risk post-vaccination, with a 95% confidence interval ranging from about 0.82 to 1.30. Given the results of the systematic review, a prospective study might require a large sample size to detect any small differences that exist, given the already low odds ratio.

This systematic review’s methodology exhibited certain limitations, primarily in its scope and the nature of the included studies. Despite a comprehensive search across multiple databases and a meticulous selection process, the review was constrained to English-language articles, potentially omitting relevant studies published in other languages. This language restriction might have led to a selection bias, limiting the generalizability of the findings. Additionally, the included studies were predominantly cohort studies, with one high-quality study and others rated as medium or low-quality. This variation in study quality could have implications for the overall reliability and robustness of the conclusions drawn. Furthermore, this review focused on specific vaccines and defined preterm birth as births before 37 weeks of gestation, which may not capture the nuances of varying gestational periods or vaccine types. The limitations of this systematic review are further compounded by numerous confounding factors that were not fully accounted for. Key among these is the influence of different viral variants and the varying phases of the pandemic, which could significantly impact the outcomes of the studies. Additionally, this review did not thoroughly consider the effect of the number of vaccine doses, the specific type of vaccine administered, and other critical factors such as comorbidities, age, ethnicity, socioeconomic status, and parity.

## 5. Conclusions

This systematic review provides essential insights into the effects of COVID-19 vaccination during pregnancy. Key findings indicated that COVID-19 vaccination does not significantly increase the risk of preterm birth, but also indicated no significant risk of adverse pregnancy outcomes, or neonatal complications. This evidence is crucial for public health policy and reassures both healthcare providers and expectant mothers about the safety of COVID-19 vaccination during pregnancy.

## Figures and Tables

**Table 1:** Study characteristics.

Study & Author	Country	Publication Year	Study Design	Study Quality
1 [21] Beharier et al.	Israel	2021	Prospective cohort	Medium
2 [22] Citu et al.	Romania	2022	Prospective cohort	Low
3 [23] Dick et al.	USA	2022	Retrospective cohort	Medium
4 [24] Goldshtein et al.	Israel	2022	Prospective cohort	High
5 [25] Rottenstreich et al.	Israel	2022	Retrospective cohort	Medium
6 [26] Theiler et al.	USA	2021	Retrospective cohort	Medium

**Table 2:** Characteristics of patients.

Study Number	Number of Patients	Age (Mean/Median)	Comorbidities	Comparison Group	Patient Characteristics
1 [21] Beharier et al.	1094 (94 unvaccinated with previous COVID-19, and 895 unvaccinated without prior SARS-CoV-2 infection)	28.8 years (COVID-19 group) vs. 31.7 years (no COVID-19 group)	DM 5.4% (COVID-19 group) vs. 8.7% (no COVID-19 group)	213 mother-child pairs (65 PCR positive, 86 vaccinated, 62 unvaccinated noninfected)	Societal groups: ~75% Jewish, ~20% Arab, ~5% other
2 [22] Citu et al.	835 (Vaccinated: 227, Unvaccinated: 608)	Vaccinated: 29.8 years, Unvaccinated: 31.2 years	Obesity (Vaccinated: 19.6%, Unvaccinated: 22.4%)	173 seronegative vs. 54 seropositive	Rural origin: 28.9% vaccinated vs. 62.1% vaccinated, Multiparous vs. primiparous: 41.6% vaccinated vs. 59.4% vaccinated), History of abortion (Vaccinated: 17.9%)
3 [23] Dick et al.	5618 (2305 vaccinated vs. 3313 unvaccinated)	30 years (matched in both groups)	Hypertensive Disorder of Pregnancy: 25 (1.1%); Gestational DM: 222 (9.6%)	Unvaccinated group, Pregnancy trimester groups	Primiparous 1 (0.3%); Nulliparous: 611 (26.5%); Smoking: 79 (3.4%)
4 [24] Goldshtein et al.	24,288 (Pre-IPTW Post-IPTWa unvaccinated—7591; Post-IPTW unvaccinated—7452; vaccinated 738)	Average age: 31.61 years	Obesity: 1768 (10.6%); Infertility: 304 (1.8%); Cancer: 168 (1.0%); Hypertension: 159 (1.0%); CKD: 118 (0.7%); Diabetes: 145 (0.9%); Cardiovascular Disease: 8 (<0.1%)	Unvaccinated group, IPTW groups	Nulliparous: 32.7% Jewish (Secular 63.9%, Arab 10.9%); Socioeconomic higher in vaccinated patients; Smoking: 798 (4.8%)
5 [25] Rottenstreich et al.	1775 (1063 unvaccinated vs. 712 vaccinated)	Vaccinated: 30.6 years; Unvaccinated: 29.5 years	Hypertensive Disorders: Vaccinated 1.4%, Unvaccinated 1.8%; Diabetes: Vaccinated 6.3%, Unvaccinated 4.2%; Obesity (BMI ≥ 30 kg/m^2^): Vaccinated 14.2%, Unvaccinated 13.2%	Unvaccinated group	Previous miscarriages: vaccinated 33.7%, unvaccinated 27.8%; previous caesarean delivery: vaccinated 16.4%, unvaccinated 12.9%; fertility treatments: vaccinated 4.6%, unvaccinated 2.3%
6 [26] Theiler et al.	2002 (1862 vaccinated vs. 140 unvaccinated)	Vaccinated: 31.8 years; Unvaccinated: 30.1 years	Pregestational diabetes mellitus: 1.4%; Pregestational hypertension: 4.3%; Asthma: 10.7%	Unvaccinated group	Education > 16 years: 46.6% in vaccinated patients; Smoking: 0% in vaccinated; Infertility treatment: 4.3% in vaccinated; Gravidity: 1 (40%) in vaccinated; Pre-pregnancy BMI: <25 in 56.5% of vaccinated patients

DM—Diabetes Mellitus; IPTW—Inverse Probability of Treatment Weights; CKD—Chronic Kidney Disease; BMI—Body Mass Index.

**Table 3:** Newborn features and vaccination characteristics.

Study Number	Vaccine Type *	Number of Doses	Time of Vaccination	Immune Response	Newborn Features
1 [21] Beharier et al.	BNT162b2	2 doses	Median 34.5 weeks GA	Strong maternal IgG response, crossing placenta; lower IgG transfer ratio for third-trimester infections; no significant differences in maternal-neonatal serological correlations between SARS-CoV-2 infected and vaccinated groups	NR
2 [22] Citu et al.	BNT162b2 and Ad26.COV2.S	1–2 doses	3rd trimester (>27 weeks)	Spike antibodies before vaccination 0.41 U/mL (seronegative) vs. 145 U/mL (seropositive), at 4 months 1083 U/mL (seronegative) vs. 10,759 U/mL (seropositive)	GW 3149 g (vaccinated) vs. 3207 g (unvaccinated)APGAR <7 at 5 min 6.3% (vaccinated) vs. 6.6% (unvaccinated)
3 [23] Dick et al.	BNT162b2 and mRNA-1273	NR	2nd and 3rd trimester	NR	Birthweight: 3280 (2980, 3590) g; APGAR 5 min < 7: 42 (1.8%); Umbilical arterial pH < 7.1: 89 (7.4%)
4 [24] Goldshtein et al.	BNT162b2	1–2 doses	1st, 2nd, and 3rd trimester	NR	49% Female; 96% Born at ≥37 Weeks’ Gestation; Follow-up: Median 126 Days (76–179) in Exposed, 152 Days (88–209) in Unexposed
5 [25] Rottenstreich et al.	BNT162b2	2 doses	3rd trimester (>27 weeks)	NR	Birthweight: vaccinated 3317.8 g, unvaccinated 3339.5 g; male gender: vaccinated 49.6%, unvaccinated 50.7%; APGAR score ≤ 7 at 1 min: vaccinated 4.2%, unvaccinated 4.6%; APGAR score ≤ 7 at 5 min: vaccinated 2.9%, unvaccinated 2.5%;
6 [26] Theiler et al.	BNT162b2, Ad26.COV2.S, and mRNA-1273	>1 dose	Median 32 weeks GA	NR	Gestational Age at Delivery: ≥37 0/7 weeks: 90.7%; Low Birthweight (<2500 g): 7.9%; Very Low Birthweight (<1500 g): 2.1%;

NR—Not Reported; *—Janssen = Ad26.COV2.S, Pfizer = BNT162b2, Moderna = mRNA-1273; GA—Gestational Age; GW—Gestational Weight.

**Table 4:** Outcomes and risk assessment.

Study Number	Pregnancy Complications	Neonatal Complications	Preterm Birth Risk (OR/RR/HR)	Other Risks (OR/RR/HR)
1 [21] Beharier et al.	Preterm birth—0.6%Other adverse outcomes—0.2%	No illness post-childbirth	0.57 (0.16–2.06)	Adverse pregnancy outcomes—2.87 (0.33–25.09)
2 [22] Citu et al.	Preterm birth—2.9%	SGA—0.9%	1.16 (0.64–2.09)	Adverse neonatal outcomes—0.96 (0.50–1.85); SGA—0.71 (0.30–1.69)
3 [23] Dick et al.	Preterm birth: 127 (5.5%);	SGA: 142 (6.2%); Cesarean Delivery: 358 (15.5%); Postpartum Hemorrhage: 79 (3.4%); Intrauterine fetal demise: 20 (0.87%); Hypertensive disorder of pregnancy: 25 (1.1%); Gestational diabetes: 222 (9.6%)	Preterm birth in 2nd Trimester vaccinated: OR 1.49 (1.11, 2.01); Preterm birth in 3rd Trimester vaccinated: OR 0.49 (0.34, 0.71);	SGA in 2nd trimester vaccinated: OR 0.73 (0.52, 1.03); SGA in 3rd trimester vaccinated: OR 0.85 (0.64, 1.13)
4 [24] Goldshtein et al.	Preterm birth: 6.1% Pre-IPTW unvaccinated vs. 7.9% vaccinated; 6.6% Post-IPTW unvaccinated vs. 6.2% vaccinated	Low birth weight: 5.8% Pre-IPTW unvaccinated vs. 7.6% vaccinated; SGA: 6.8% Pre-IPTW unvaccinated vs. 7.5% vaccinated;	0.95 (0.83–1.10)	SGA: 0.97 (0.87–1.08); Congenital anomalies: 0.69 (0.44–1.04); All-cause neonatal hospitalizations: 0.99 (0.88–1.12); Post-neonatal hospitalizations: 0.95 (0.84–1.07); Infant mortality: 0.84 (0.43–1.72); Major heart malformations: 0.46 (0.24–0.82)
5 [25] Rottenstreich et al.	Preterm birth: 4.4%; elective caesarean delivery: vaccinated 11.5%, unvaccinated 7.6%; vacuum-assisted delivery: vaccinated 3.2%, unvaccinated 6.2%; postpartum hemorrhage: vaccinated 7.3%, unvaccinated 10%;	Intrauterine fetal death: vaccinated 0.7%, unvaccinated 0.5%; NICU admission: vaccinated 4.1%, unvaccinated 4.5%	1.01 (0.62–1.63)	COVID-19 vaccination not associated with maternal composite adverse outcome: 0.8 (0.61–1.03); composite adverse neonatal outcome: vaccinated 7.9%, unvaccinated 11.4%; significantly reduced risk with vaccination: 0.5 (0.36–0.74)
6 [26] Theiler et al.	Preterm birth: 3.5%;Adverse outcomes: vaccinated 5.0%, unvaccinated 4.9%; eclampsia or preeclampsia: 0.7% in vaccinated; gestational hypertension: 13.6% in vaccinated	C-section 9.9%; Postpartum hemorrhage 0.1%; Stillbirth 2.9%; No significant differences in neonatal outcomes	1.09 (0.63–1.86)	C-section—1.05 (0.82–1.34); Postpartum hemorrhage—2.66 (0.31–22.61); Stillbirth—1.02 (0.06–18.01)

OR—Odds Ratio; RR—Risk Ratio; HR—Hazard Ratio; SGA—Small for Gestational Age; NICU—Neonatal Intensive Care Unit.
